# Schizophrenia risk proteins ZNF804A and NT5C2 interact in cortical neurons

**DOI:** 10.1111/ejn.16254

**Published:** 2024-01-26

**Authors:** Afra Aabdien, Laura Sichlinger, Zoe Borgel, Madeleine R. Jones, Iain A. Waston, Nicholas J. F. Gatford, Pooja Raval, Lloyd Tanangonan, Timothy R. Powell, Rodrigo R. R. Duarte, Deepak P. Srivastava

**Affiliations:** ^1^ Department of Basic and Clinical Neuroscience, The Maurice Wohl Clinical Neuroscience Institute, Institute of Psychiatry, Psychology & Neuroscience King's College London London UK; ^2^ MRC Centre for Neurodevelopmental Disorders, Institute of Psychiatry, Psychology & Neuroscience King's College London London UK; ^3^ Social, Genetic & Developmental Psychiatry Centre, Institute of Psychiatry, Psychology & Neuroscience King's College London London UK

**Keywords:** dendrite, dendritic spine, NT5C2, psychosis, synapse, ZNF804A

## Abstract

The zinc finger protein 804A (*ZNF804A*) and the 5′‐nucleotidase cytosolic II (*NT5C2*) genes are amongst the first schizophrenia susceptibility genes to have been identified in large‐scale genome‐wide association studies. ZNF804A has been implicated in the regulation of neuronal morphology and is required for activity‐dependent changes to dendritic spines. Conversely, NT5C2 has been shown to regulate 5′ adenosine monophosphate‐activated protein kinase activity and has been implicated in protein synthesis in human neural progenitor cells. Schizophrenia risk genotype is associated with reduced levels of both *NT5C2* and *ZNF804A* in the developing brain, and a yeast two‐hybrid screening suggests that their encoded proteins physically interact. However, it remains unknown whether this interaction also occurs in cortical neurons and whether they could jointly regulate neuronal function. Here, we show that ZNF804A and NT5C2 colocalise and interact in HEK293T cells and that their rodent homologues, ZFP804A and NT5C2, colocalise and form a protein complex in cortical neurons. Knockdown of the *Zfp804a* or *Nt5c2* genes resulted in a redistribution of both proteins, suggesting that both proteins influence the subcellular targeting of each other. The identified interaction between ZNF804A/ZFP804A and NT5C2 suggests a shared biological pathway pertinent to schizophrenia susceptibility within a neuronal cell type thought to be central to the neurobiology of the disorder, providing a better understanding of its genetic landscape.

## Graphical abstract

Recent studies indicate a physical interaction between schizophrenia‐associated genes *ZNF804A* and *NT5C2*. This study reveals that these proteins co‐localise and interact in HEK293 cells and primary rat cortical neurons. Knockdown experiments reveal mutual influences on subcellular targeting, and may indicate that these two proteins function as a complex to regulate neuronal processes.

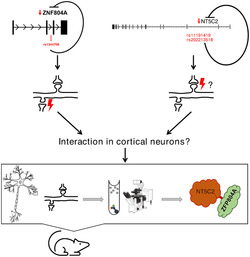

**Abbreviations**
AMPK: adenosine monophosphate kinase
APV: DL‐2‐amino‐5‐phosphonovaleric acid
AU: arbitrary units
cLTP: chemical long‐term potentiation
co‐IP: co‐immunoprecipitation
DAPI: 4′,6‐diamidino‐2‐phenylindole
DIV: day in vitro
DMEM: Dulbecco's modified Eagle medium: F12
dsDNA: double stranded deoxyribonucleic acid
E: embryonic day
EDTA: ethylenediaminetetraacetic acid
eEF2: eukaryotic elongation factor 2
EtOH: ethanol
FBS: foetal bovine serum
GFP: green fluorescent protein
GluN1: glutamate ionotropic *N*‐methyl‐D‐aspartate receptor subunit NR1
GWAS: genome‐wide association studies
HBSS: Hanks' balanced salt solution
HEK293T: human embryonic kidney cells expressing mutants of SV40 large T antigen
HRP: horse radish peroxidase
IgG: immunoglobulin
IP: immunoprecipitation
kDa: kilodaltons
MAP 2: microtubule associated protein 2
MgCl_2_
: magnesium chloride
mRNA: messenger ribonucleic acid
mTOR: mammalian target of rapamycin
NaCl: sodium chloride
NGS: normal goat serum
NT5C2: cytosolic 5′‐nucleotidase II
P: precipitate
PBS: phosphate‐buffered saline
PDL: poly‐D‐Lysine
PSD‐95: postsynaptic density protein‐95
*r*
: Pearson's coefficient
RPM: revolutions per minute
S.E.M.: standard error of the mean
S: supernatant
SDS: sodium dodecyl sulfate
siRNA: small interfering ribonucleic acid
SNP: single nucleotide polymorphism
SV2A: synaptic vesicle glycoprotein 2A
TBS: tris‐buffered saline
TBS‐T: tris‐buffered saline Tween20
tRNA: transfer ribonucleic acid
Tx/RIPA: TritonX‐100 and RIPA
VGLUT: vesicular glutamate transporter 1
ZFP804A: zinc finger protein 804A rat homologue
ZNF804A: zinc finger protein 804A

## 1 INTRODUCTION

Schizophrenia is a chronic psychiatric disorder with a complex aetiology and a substantial underlying genetic component (Birnbaum & Weinberger, 2017; Lewis & Levitt, 2002; Rapoport et al., 2012; Smeland et al., 2020). Genome‐wide association studies (GWAS) suggest that one‐third of the variability in schizophrenia is attributed to common genetic variants (Ripke et al., 2011). Recent studies suggest as many as 270 independent loci involved in schizophrenia risk (Ripke et al., 2020; Trubetskoy et al., 2022), emphasizing the intricate genetic landscape underlying this psychiatric disorder. However, there are many gaps in knowledge regarding the function of these genes and how they may interact. Ultimately, advancing our understanding of the function of specific risk genes in relevant models has the potential to improve our current understanding of this disorder, to help pave the way for the development of novel diagnostic and therapeutic options.

The zinc finger protein 804A (*ZNF804A*) and the 5′‐nucleotidase, cytosolic II (*NT5C2, cN‐II*) loci, were among the first to be identified in early GWAS of schizophrenia (O'Donovan et al., 2008; Ripke et al., 2011), and they remain robustly associated with this disorder in more recent studies (Trubetskoy et al., 2022). They are also associated with susceptibility to multiple psychiatric disorders combined (Amare et al., 2019; Cross‐Disorder Group of the Psychiatric Genomics Consortium, 2013; Williams et al., 2011), suggesting that translating association signals from these regions into neurobiological risk mechanisms can help advance understanding of psychiatric disorders in general. Recently, an interactome study using a yeast two‐hybrid assay indicated that ZNF804A interacts with multiple proteins, including NT5C2 (Zhou et al., 2018). Of these putative interacting partners, only NT5C2 is associated with schizophrenia susceptibility. Previous work from our group showed that schizophrenia risk genotype at the 10q24 locus is associated with downregulation of *NT5C2* during neurodevelopment and in the adult brain (Duarte et al., 2016). Schizophrenia risk genotype at the ZNF804A locus was also associated with downregulation of this gene, although predominantly in the developing brain (Hill & Bray, 2012). These data led us to hypothesise that such shared pattern of downregulation in the developing brain could indicate a converging regulatory pathway related to schizophrenia that could be relevant to neuronal function.

The function of both proteins has been extensively investigated by our group and others. For example, ZNF804A protein is expressed in the human cerebral cortex, particularly in pyramidal neurons (Dong et al., 2021; Tao et al., 2014), and it has been shown to regulate neurite outgrowth in developing human and rodent neurons (Deans et al., 2017; Dong et al., 2021). In mice, ZFP804A (rodent homologue of ZNF804A) has been found to regulate migration of developing neurons in embryonic brains (Zhou et al., 2018). ZNF804A has also been found to localise to putative synapses, as demonstrated by co‐localisation with the synaptic proteins PSD‐95 and GluN1 (Deans et al., 2017). It was also found to be enriched in synaptic fractions and to localise to dendritic spines, where the majority of excitatory synapses occur in the mammalian brain (Deans et al., 2017). Consistent with a role for the encoded zinc finger protein at synapses, knockdown or knockout of *Zfp804a* resulted in a reduction of dendritic spine density (Deans et al., 2017; Dong et al., 2021; Huang et al., 2020), whereas an increase in spine density was observed when the entire length or shorter disease‐associated isoform of the protein was overexpressed in cortical neurons (Dong et al., 2021; Zhou et al., 2020). Moreover, activity‐dependent remodelling of dendritic spines was impaired in cortical neurons with reduced *ZNF804A* expression levels (Deans et al., 2017). The NT5C2 protein, on the other hand, also appears to be enriched in cortical neurons (Duarte et al., 2019), but less is known about its neurological function, relative to ZNF804A. Knockdown of *NT5C2* in human neural progenitor cells is associated with increased phosphorylated 5′ adenosine monophosphate‐activated protein kinase (AMPK), which negatively regulates mammalian target of rapamycin (mTOR) and eukaryotic elongation factor 2 (eEF2), suggesting that NT5C2 plays a role in protein translation (Duarte et al., 2019). A neuronal knockdown of *CG32549* (the *Drosophila melanogaster* homologue) was associated with motor defects (Duarte et al., 2019) and increased sleep in fly (Singgih et al., 2021).

In our study, we assessed the subcellular localisation of ZNF804A and NT5C2 and explored whether they formed a protein complex in heterologous HEK293T cells and rodent cortical neurons. Next, we investigated whether a small interfering RNA (siRNA)‐mediated knockdown of either protein resulted in the altered presence of the other protein at synapses. Ultimately, we provide data that suggests that two schizophrenia risk genes function as a complex to regulate synaptic function.

## 2 RESULTS

### 2.1 ZNF804A and NT5C2 participate in a protein complex when expressed in HEK293 cells

**FIGURE 1:**
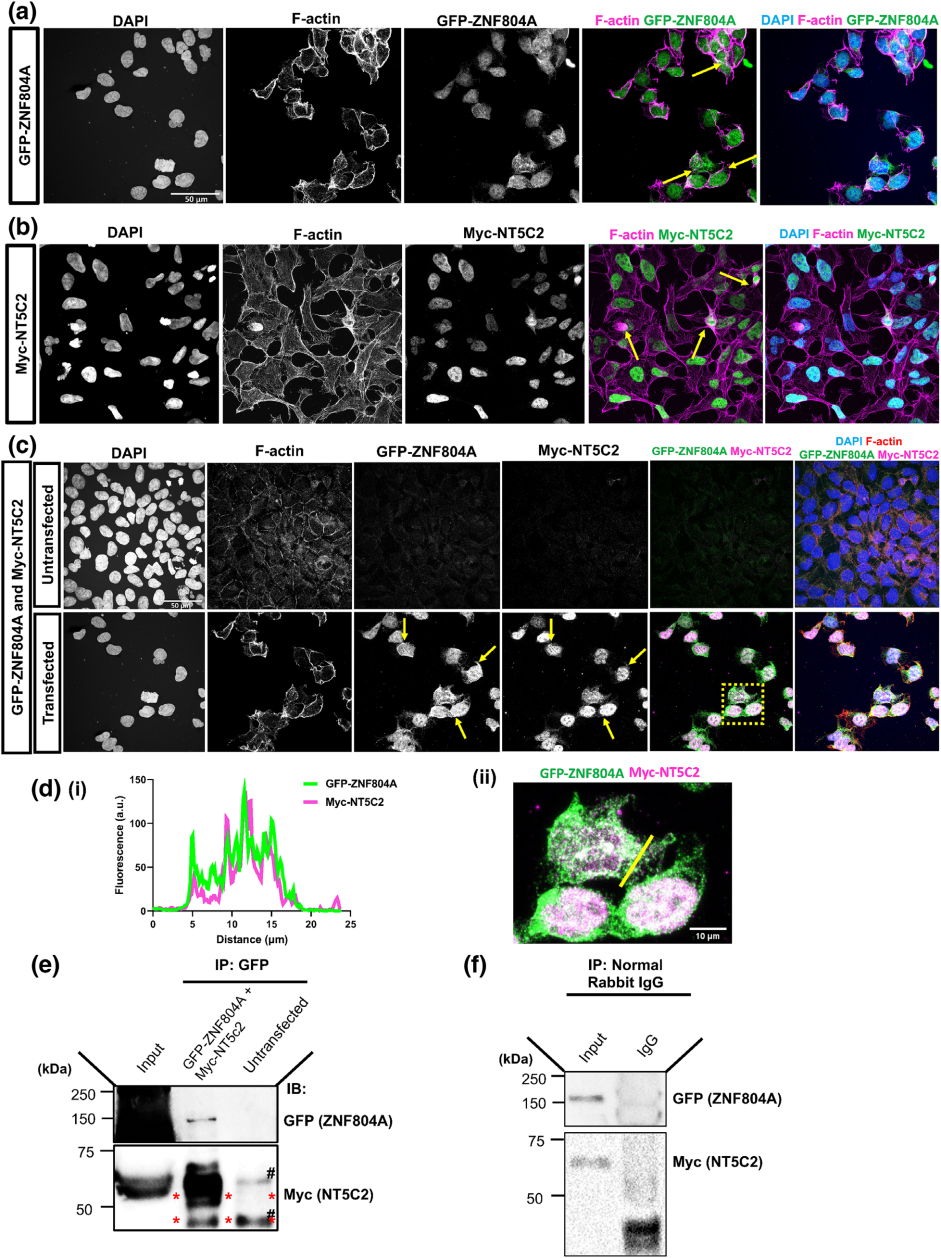
NT5C2 and ZNF804A form a protein complex and co‐localise when expressed in HEK293 cells. (a) Representative confocal image of HEK293T cells expressing GFP‐ZNF804A and co‐stained for F‐actin. Ectopically expressed GFP‐ZNF804A localises to cell cytoplasm and plasma membrane (yellow arrows). Near the plasma membrane, GFP‐ZNF804A co‐localises with F‐actin. (b) Representative confocal image of HEK293T cells expressing Myc‐NT5C2 and co‐stained for F‐actin. Ectopically expressed Myc‐NT5C2 localises to cell cytoplasm (yellow arrows) like previous descriptions. (c) Confocal images of untransfected HEK293T cells, or cells co‐transfected with GFP‐ZNF804A and Myc‐NT5C2. In co‐expressing cells, GFP‐ZNF804A and Myc‐NT5C2 co‐localise to the cell cytoplasm and near the plasma membrane (yellow arrows). Yellow dotted box indicates area magnified in (d). (d) (i) + (ii) Intensity plot and magnified image of dotted box from (c), demonstrating co‐localisation of GFP‐ZNF804A and Myc‐NT5C2. (e) Co‐immunoprecipitation (co‐IP) assay of HEK293T cells co‐expressing GFP‐ZNF804A and Myc‐NT5C2. Cell lysates were immunoprecipitated with a Myc antibody to isolate NT5C2 and the interacting partner. Samples were subjected to immunoblotting and then subsequently probed with antibodies for Myc (to detect Myc‐NT5C2) and GFP (to detect GFP‐ZNF804A). In the lower blot, ‘#’ denotes non‐specific IgG bands, and the red ‘*’ indicates Myc‐NT5C2. A specific band for GFP‐ZNF804A was detected in immunoprecipitated cell lysates expressing both proteins but not from untransfected cell lysate. (f) Co‐IP assay of HEK293T cells co‐expressing GFP‐ZNF804A and Myc‐NT5C2, whereby cell lysates were incubated with control normal rabbit IgG. Scale bar = 50 μm for (a) and (b) and 10 μm for (d).

As a previous yeast‐two hybrid assay has suggested that ZNF804A and NT5C2 are interacting partners (Zhou et al., 2018), we were interested in confirming this interaction in situ. We first examined the localisation of ectopically expressed GFP‐ZNF804A and Myc‐NT5C2 in HEK293T cells. Consistent with findings from Deans et al. (2017), GFP‐ZNF804A localised to both nuclear, cytosolic and membrane compartments (Figure 1a). HEK293T cells transfected with Myc‐NT5C2 revealed that this protein localised to the nucleus as well as the cytosol (Figure 1b). This is contrary to previous findings from Duarte et al. (2019) that showed a predominate localisation of MNT5C2 to the cytosol of human progenitor cells. This difference could be due to the use of an overexpression system in non‐neuronal cells. Next, we co‐transfected GFP‐ZNF804A and Myc‐NT5C2 into HEK293T cells and observed that GFP‐ZNF804A and Myc‐NT5C2 co‐localised in the nucleus, cytoplasm and near the plasma membrane of HEK295T cells (Figure 1c). This was further exemplified by a line graph construction, whereby the fluorescence intensity of both proteins was measured (Figure 1d, i and ii), and the overlap between the intensities provided evidence of co‐localisation.

To validate these findings and determine whether ZNF804A and NT5C2 formed a protein complex, we performed a co‐immunoprecipitation using HEK293T cells ectopically expressing both proteins. Cells co‐transfected with GFP‐ZNF804A and Myc‐NT5C2 were immunoprecipitated with an anti‐Myc antibody to isolate NT5C2 and its interacting partners. As expected, Myc‐NT5C2 was readily detected in the immunoprecipitation (IP) sample (Figure 1e, lower blot; red ‘**’ denotes Myc‐NT5C2) and not in the normal rabbit IgG sample (Figure 1f). In addition, GFP‐ZNF804A was also present in the IP sample, indicating that ZNF804A and NT5C2 were part of a protein complex (Figure 1e). Taken together, these data suggest that ZNF804A and NT5C2 co‐localise form a protein complex in HEK293T cells.

### 2.2 ZNF804A and NT5C2 are expressed within multiple subcellular compartments in cortical neurons

**FIGURE 2:**
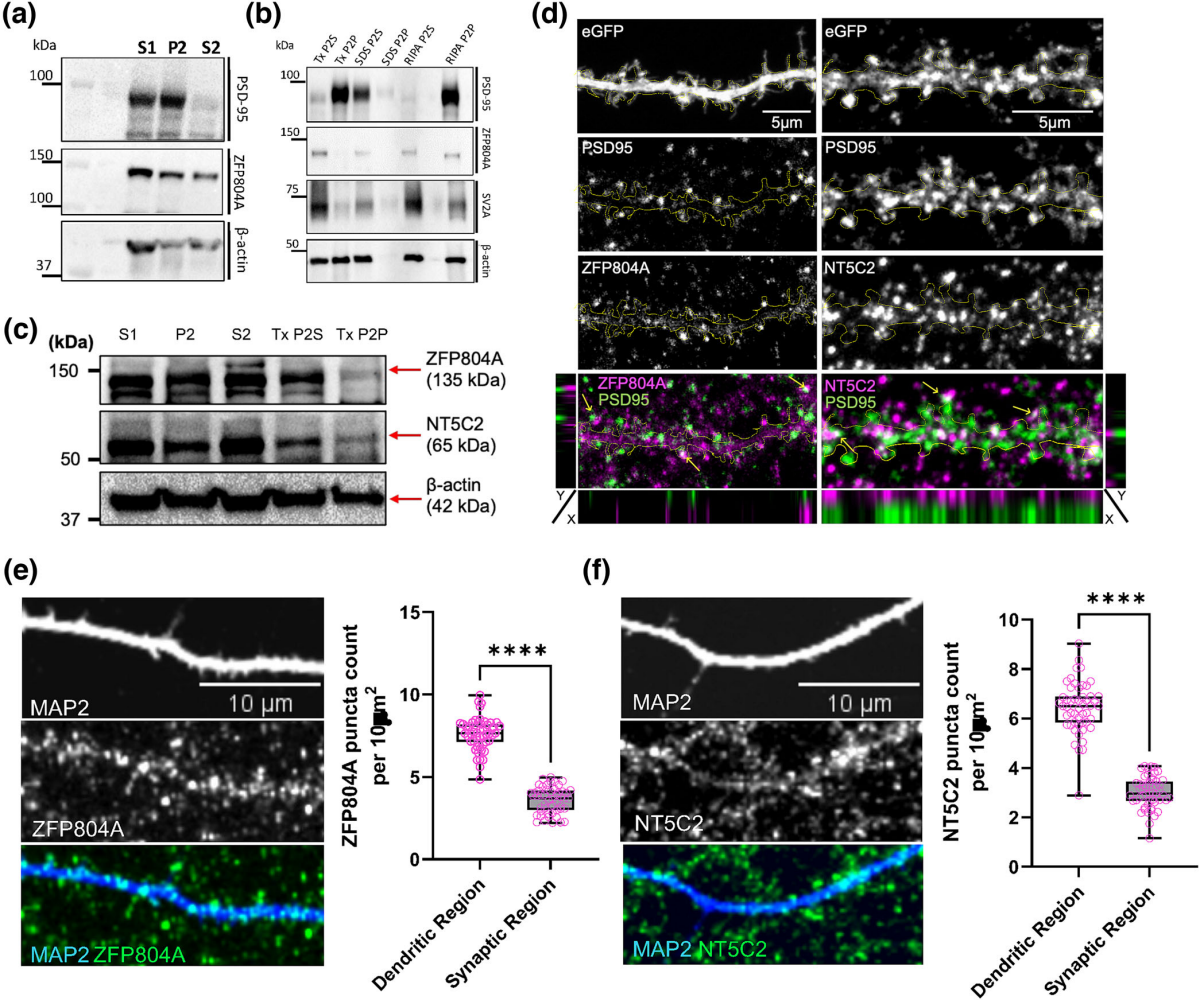
Subcellular localisation of ZFP804A and NT5C2 in cortical neurons. (a–c) Western blotting of cell fractions generated from mouse cortex. S1, extranuclear cell lysate; S2, cytosol; P2, crude membrane/synaptic fraction; P2S, membrane/synaptic supernatant, P2P, membrane/synaptic precipitate. (a) Immunoblotting of cortical cell fractionations for PSD‐95 and ZFP804A with β‐actin loading control. PSD‐95 was enriched in the P2 fractions, while ZFP804A was present in all fractions but enriched in S2 cytosolic fraction. (b) Immunoblotting of detergent treated crude synaptic fractions of mouse cortex, for ZFP804A, PSD‐95 and SV2A with a β‐actin loading control. PSD‐95 was enriched in the Tx P2P and RIPA P2P fractions, while SV2A and ZFP804A were predominately present in Tx P2S and RIPA P2S soluble fractions. (c) Immunoblotting of cortical cell fractionations for ZFP804A and NT5C2 with a β‐actin loading control. ZFP804A and NT5C2 were present in all fractions; ZFP804A and NT5C2 were abundant in S2 fractions compared to P2. ZFP804A and NT5C2 were present more in TritonX‐100 soluble fractions (Tx P2S) to TritonX‐100 insoluble fractions (Tx P2P). PSD‐95 was used to demonstrate synaptic enrichment. (d) Representative confocal image of a section of dendrite from DIV20 cortical neurons transfected with eGFP constructs and co‐stained for PSD‐95, ZFP804A and NT5C2. Yellow arrows indicate the localisation co‐localisation of ZFP804A and NT5C2 with PSD‐95 along the synapses. Further confirmation was also conducted with orthogonal analysis. (e) Representative confocal image of a section of dendrite from DIV20 cortical neurons immunostained for MAP 2 (morphological marker) and a previously validated antibody for ZFP804A. Quantification of ZFP804A puncta across 10 μm^2^ in dendrites or crude synaptic region is shown in a box and whisker plot, showing maximum and minimum values. (f) Representative confocal image of a section of dendrite from DIV20 cortical neurons immunostained for MAP 2 (morphological marker) and NT5C2. Quantification of NT5C2 puncta across 10 μm^2^ in dendrites or crude synaptic region is shown in a box and whisker plot, showing maximum and minimum values. *n* = 51 cells from three independent experiments.

Previous work has demonstrated that ZNF804A localises to somatodendritic compartments of neurons, particularly in dendritic spines (Deans et al., 2017). In comparison, little is known about the distribution and organisation of NT5C2 in neurons. We first sought to confirm ZFP804A distribution in vivo, using cell fractions generated from the mouse cortex (Figure S1). Cortices were subjected to subcellular fractionation, yielding the whole cell (non‐nuclear) (S1), cytosolic (S2) and crude synaptic (P2) subcellular fractions (Jones et al., 2014). It is of note that the preparation of the P2 fraction will also include membranes from other organelles, including mitochondria. However, owing to the presence of the synaptic protein PSD‐95 within this fraction, we refer to the P2 fraction as a crude synaptic fraction. Consistent with our previous finding (Deans et al., 2017), ZFP804A was present in both cytosolic and crude synaptic fractions (Figure 2a). To assess the association of ZFP804A with synaptic membranes, crude synaptic fractions were further subjected to Triton‐X100, SDS or RIPA detergent extraction to produce a supernatant (S) and precipitate (P) (Figure 2b). As expected, PSD‐95, a significant component of synapses and the post‐synaptic density, was present in the precipitate of TritonX‐100 and RIPA detergent synaptic fractions (Tx/RIPA P2P), indicating a tight association with synaptic membranes (Figure 2b). Conversely, SV2A, a pre‐synaptic vesicle protein, was present in soluble fractions following detergent extraction (Figure 2b). ZFP804A was also found to be predominately present in soluble fractions (P2S) following detergent extraction indicating a loose association with synaptic membranes (Figure 2b). Examination of NT5C2 distribution revealed that it was abundantly present in cytosolic (S2) fractions and within crude synaptic fractions to a lesser extent (Figure 2c). Within the P2 fraction, NT5C2 was only loosely associated with synaptic membranes, as indicated by the presence of the protein in the supernatant fraction (P2S) following detergent extraction (Figure 2c). These findings corroborate that ZFP804A/ZNF804A localises to multiple subcellular compartments beyond the nucleus (Deans et al., 2017) and suggest that NT5C2 is predominately present in the cytosol of cortical neurons.

To further investigate the subcellular localisation of ZFP804A and NT5C2 across dendrites and synaptic regions, we performed a series of immunostaining experiments in DIV20 (days in vitro) rat primary cortical neurons. Rat primary cortical neurons were transfected with eGFP constructs to highlight neuronal morphology and co‐stained with either ZFP804A or NT5C2 as well as PSD‐95. ZNF804A could be observed as punctate structures along dendrites. In addition, a subset of dendritic spines were also positive for ZFP804A (Figure 2d). ZFP804A also co‐localised with PSD‐95 (Figure 2d). ZFP804A was also seen to co‐localise with the excitatory pre‐synaptic marker VGluT1 (Figure S2A), supporting the presence of this protein at excitatory synapses. NT5C2 was also present within dendrites and to a lesser extent, within dendritic spines, where it showed limited co‐localisation with PSD‐95 and VGluT1 (Figures 2 and S2). These data indicate that ZFP804A and NT5C2 localise to both dendrites and synaptic regions, suggesting that both proteins are present within the same subcellular compartments in neurons.

To confirm our findings of the presence of either ZFP804A and NT5C2 at synapses or dendrites, we quantified the puncta across an area of 10 μm^2^ across the dendrite. In this analysis, we defined our region of synaptic interest as the region occupied by 2 μm on either side of the dendrite, thus capturing both pre‐ and post‐synaptic regions. Staining for endogenous ZFP804A revealed that the protein was present more in the dendritic region compared to the crude synaptic region (*p* < 0.0001, *n* = 9) (Figure 2e). Staining for endogenous NT5C2 revealed a similar distribution, with a significant presence within the dendritic region compared to the crude synaptic region (*p* < 0.0001, *n* = 9) (Figure 2f). Taken together, these data suggest that ZFP804A and NT5C2 have a similar sub‐cellular distribution in cortical neurons, with both proteins being present along dendrites and within dendritic sines, but with ZFP804A showing a greater presence at excitatory synapses than NT5C2.

### 2.3 ZFP804A and NT5C2 colocalise and interact in cortical neurons

**FIGURE 3:**
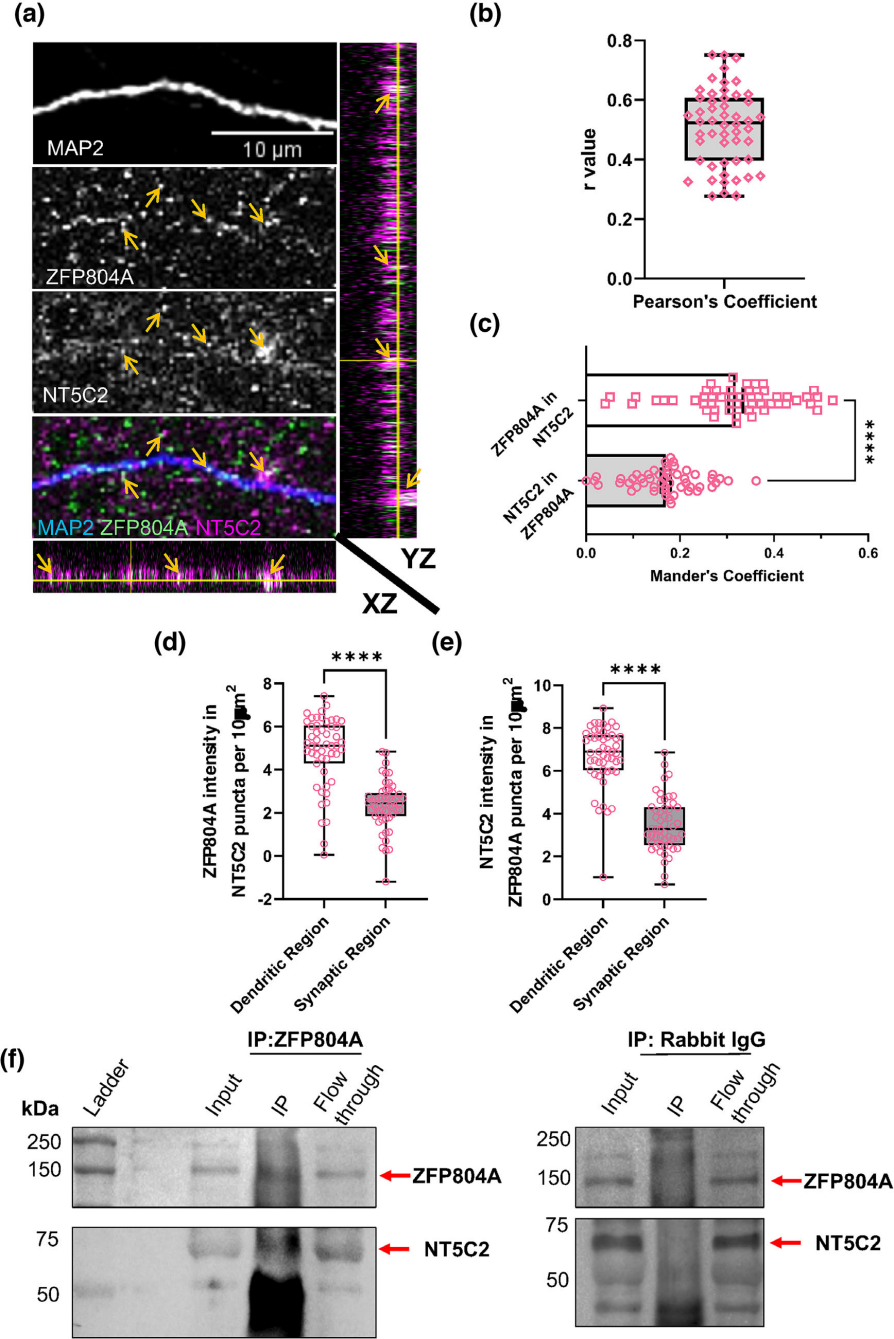
NT5C2 and ZFP804A co‐localise and form a protein complex near synapses in rat cortical neurons. (a) Representative confocal image of DIV 20 cortical neurons immunostained for MAP 2 (morphological marker), NT5C2 and ZFP804A. Orange arrows indicate the co‐localisation of NT5C2 and ZFP804A along dendrites, whereas white arrowheads indicate co‐localised puncta in synaptic regions. XZ and YZ orthogonal view further demonstrate co‐localisation of both proteins (orange arrows). (b) Pearson's coefficient (*r*) quantification for the extent of co‐localisation between ZFP804A and NT5C2 in DIV 20 primary cortical neurons. Co‐localisation of ZFP804A and NT5C2 was confirmed since *r* was >0, thus showing a positive correlation between ZFP804A and NT5C2 puncta intensity overlap. Values >0 suggest positive co‐localisation for both puncta, with significantly more ZFP804A in NT5C2 (parametric unpaired *t*‐test ****p < 0.0001), indicating a positive correlation between the overlapping intensities of ZFP804A and NT5C2 puncta across dendritic and synaptic regions; *n* = 52 cells, three biological replicates. (c) Quantification of relative intensities of ZFP804A in NT5C2 versus NT5C2 in ZFP804A across dendritic and crude synaptic regions using Mander's coefficient. Significantly more ZFP804A puncta were found in NT5C2 (parametric unpaired *t*‐test ****p < 0.0001, *n* = 51 cells, three biological replicates). (d and e) Quantification of co‐localisation: (d) density of NT5C2 puncta across an area of 10 μm^2^ positive for ZFP804A and (e) density of ZFP804A puncta positive for NT5C2 staining along dendrites or within synaptic regions. *n* = 51 cells, three biological replicates. (f) Co‐immunoprecipitation (co‐IP) assay of DIV 20 cortical neurons. Cell lysates were immunoprecipitated with either an antibody against ZNF804A/ZFP804A or against rabbit IgG (control). Samples were subjected to immunoblotting and then subsequently probed with antibodies for NT5C2 and ZNF804A. A specific band for ZFP804A was detected in the input, ZNF804A‐IP and flow through lanes as expected—no band was detected in the rabbit IgG‐IP lane, indicating the specificity of the assay. Probing with an antibody against NT5C2 revealed bands in the input, ZNF804A IP and flow through lanes, but not the rabbit IgG‐IP lane. This indicates that NT5C2 is part of a protein complex with ZNF804A.

Considering that ZNF804A and NT5C2 form a protein complex in HEK293T cells and that ZFP804A and NT5C2 localise to the same subcellular compartments in cortical neurons, we sought to explore whether these proteins were part of a protein complex in rat cortical neurons, in situ. First, we examined whether both proteins co‐localise in cortical neurons. In DIV20 cortical neurons, both ZFP804A and NT5C2 co‐localise in both dendrites and within synaptic regions: This is demonstrated by the multiple orange arrows and the green/magenta overlap in the orthogonal projections (Figure 3a). To further confirm the co‐localisation of ZFP804A and NT5C2, we calculated Pearson's coefficient (*r*) (Figure 3b). This quantification revealed *r* > 0, confirming a positive correlation between the overlap of ZFP804A and NT5C2 puncta across dendritic and synaptic regions (Pearson's coefficient [*r*]: 0.5084 ± 0.01795) (Figure 3b). Furthermore, we wanted to understand whether there was a discrepancy in the extent of co‐localisation between ZFP804A and NT5C2 using Mander's coefficient (Figure 3c). Quantification revealed significantly more ZFP804A puncta overlapping with NT5C2 puncta (Mander's coefficient: ZFP804A in NT5C2: 0.3186 ± 0.01524; NT5C2 in ZFP804A: 0.1699 ± 0.01031), *t*(7) = 8.085, *p* < 0.0001 (Figure 3c).

To investigate whether ZFP804A and NT5C2 co‐localise within the dendrite or the surrounding synaptic region, we calculated the number of ZFP804A or NT5C2 puncta that were positive for the reciprocal protein. This analysis revealed significantly more ZFP804A puncta overlapping within NT5C2 puncta in dendritic regions compared to crude synaptic regions, *t*(100) = 8.9, *p* < 0.0001, *n* = 8 cells from three independent replicates (Figure 3d). The inverse relationship was also found to be true, whereby significantly more NT5C2 puncta were found to overlap with ZFP804A puncta in synaptic regions compared to dendrites, *t*(100) = 12.12, *p* < 0.0001, *n* = 8 cells from three independent replicates (Figure 3e).

To demonstrate that this co‐localisation represented a potential interaction between the two proteins, we performed a co‐immunoprecipitation assay for ZFP804A from cortical neuron lysates. Consistent with our data from heterologous cells, immunoblotting revealed that NT5C2 was present in lysates immunoprecipitated for ZFP804A but not for rabbit IgG (Figure 3f). These data indicate that ZFP804A and NT5C2 colocalize near synaptic regions in cortical neurons and that they are part of the same protein complex.

### 2.4 Knockdown of *Zfp804a* or *Nt5c2* results in the redistribution of reciprocal protein

**FIGURE 4:**
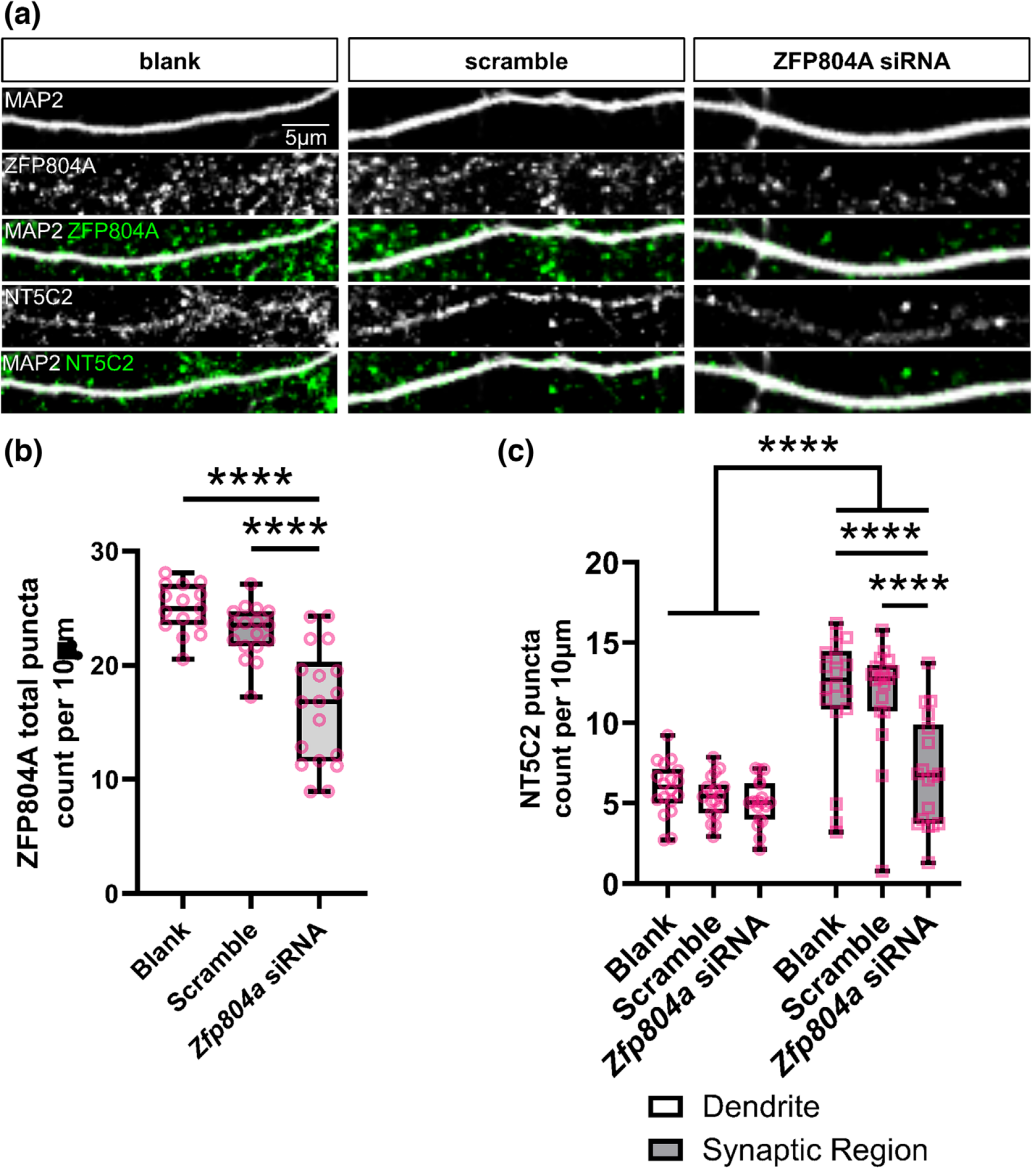
Knockdown of *Zfp804a* causes the redistribution of NT5C2. (a) Representative confocal image of DIV20 cortical neurons either untransfected (blank) or transfected with a scramble siRNA (scramble) or an siRNA for *Zfp804a* (*Zfp804a* siRNA). Cells were immunostained for MAP 2 (morphological marker), an antibody against ZFP804A (green) or NT5C2 (green). (b) Quantification of ZFP804A linear density in blank, scramble or *Zfp804a* siRNA conditions. ZFP804A linear density is shown as a box and whisker plot, with minimum and maximum values. *n* = 15–18 cells from four independent experiments. (c) Quantification of NT5C2 linear density along dendrites or crude synaptic regions, in blank, scramble or *Zfp804a* siRNA conditions. The box and whisker plot show minimum and maximum. *n* = 18 cells from four independent experiments.

To further investigate a functional interaction between ZFP804A and NT5C2 in cortical neurons, we knocked down *Zfp804a* using a previously validated siRNA (Deans et al., 2017) in DIV15 primary rat cortical neurons. We then observed the effects of this knockdown on synaptic and dendritic NT5C2 localisation at DIV20. We first validated the efficacy of the *Zfp804a* siRNA by quantifying the number of ZFP804A puncta between *Zfp804a* siRNA knockdown, scrambled siRNA or blank (untransfected) conditions. The *Zfp804a* siRNA was shown to successfully target ZFP804A as significantly fewer ZFP804A puncta were found in the siRNA knockdown condition compared to both the blank and scramble conditions—puncta per 10 μm: Blank, 22.45 ± 1.44; Scramble, 22.05 ± 1.03; *Zfp804a* siRNA, 16.38 ± 1.20. One‐way ANOVA: *F*(2, 47) = 27.04, *p* < 0.0001, Blank/siRNA Bonferroni: *t*(5) = 6.91, *p* < 0.0001, *n* = 6; Scramble/siRNA Bonferroni: *t*(5) = 5.46, *p* < 0.0001, *n* = 6 (Figure 4a+b). Furthermore, no significant difference in ZFP804A puncta was observed between the blank or scramble conditions, indicating the specificity of the siRNA treatment.

Next, we evaluated the effects of *Zfp804a* knockdown in DIV15 rat primary cortical neurons on NT5C2 puncta in the dendrites and synaptic regions of DIV20 cortical neurons. *Zfp804a* knockdown significantly reduced the density of NT5C2 puncta in synaptic regions—puncta per 10 μm; Blank, 11.78 ± 0.93; Scramble, 11.73 ± 0.81; *Zfp804a* siRNA, 6.88 ± 0.80. Two‐way ANOVA: *F*(2, 102) = 7.29, *p* = 0.0011; Bonferroni post hoc test (*p* < 0.0001, *n* = 6) (Figure 4a+c). In contrast, no effect on NT5C2 puncta number was observed in dendrites across conditions (Figure 4c).

**FIGURE 5:**
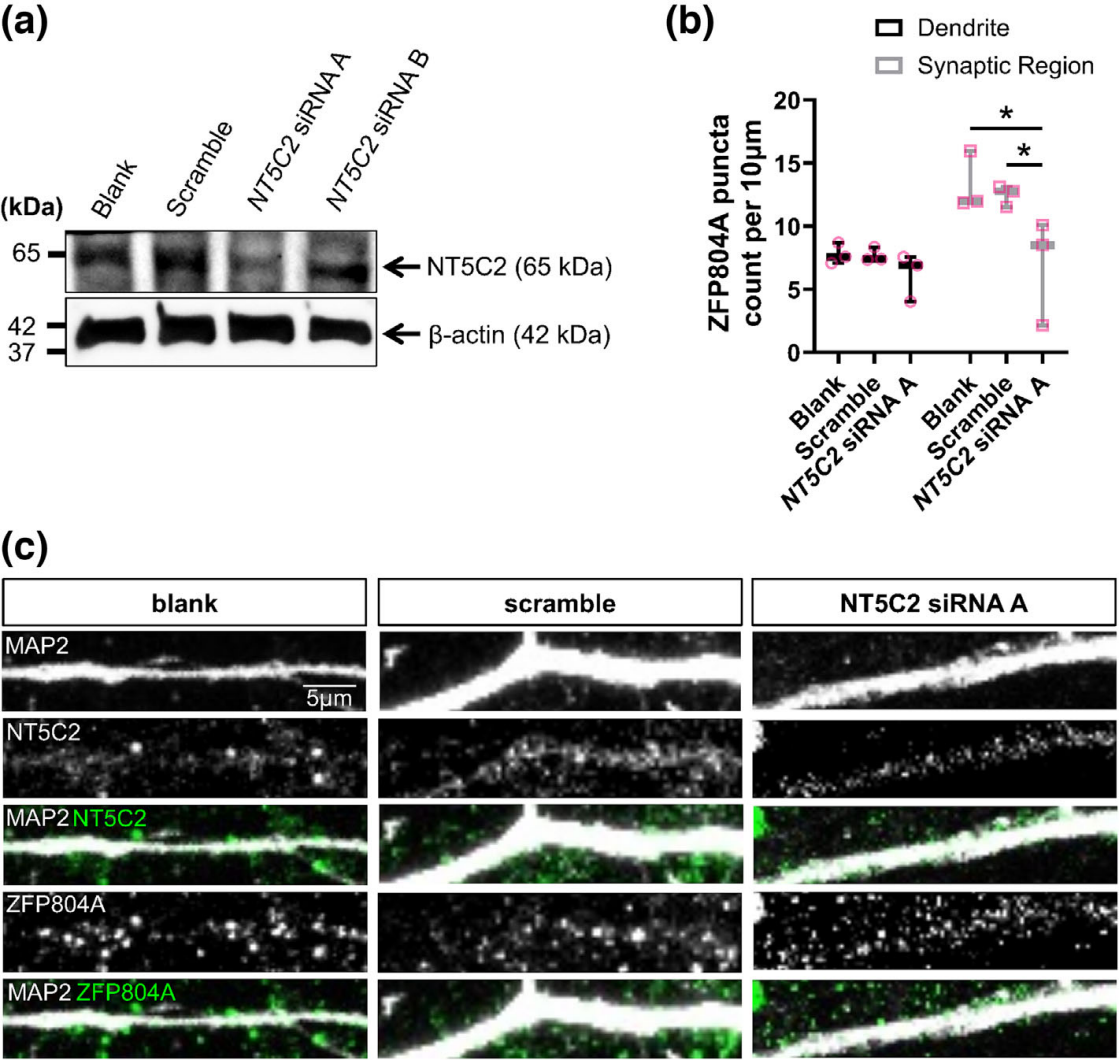
Knockdown of *Nt5c2* causes the redistribution of ZFP804A. (a) Western blot of cell lysates from DIV20 cortical neurons either untransfected (blank) or transfected with a scramble siRNA (scramble) or an siRNA (A or B) for *Nt5c2* (*Nt5c2* siRNA A or *Nt5c2* siRNA B). Immunoblots were probed with antibodies against NT5C2 or β‐actin (loading control). (b) Quantification of ZFP804A linear density in blank, scramble or *Nt5c2* siRNA A conditions. The box and whisker plot show minimum and maximum values. *n* = 15 cells from three independent experiments. (c) Representative confocal image of DIV20 cortical neurons, either untransfected (blank) or transfected with a scramble siRNA (scramble) or an siRNA against *Nt5c2* (*Nt5c2* siRNA A). Cells were immunostained for MAP 2, ZFP804A and NT5C2.

To further probe the nature of this functional interaction, we performed the inverse experiment to understand if *Nt5c2* expression can influence ZFP804A localisation. We knocked down *Nt5c2* using a siRNA previously validated (Duarte et al., 2019) in DIV15 rat primary cortical neurons. We then observed the effects of the knockdown on synaptic and dendritic ZFP804A localisation at DIV20 to allow for sufficient protein turnover. We first validated the efficacy of two siRNAs for *Nt5c2* via immunoblotting comparing siRNA A or B knockdown, scrambled siRNA or blank (untransfected) conditions. This revealed that the *Nt5c2* siRNA A successfully targeted and knocked down NT5C2 in primary rat cortical neurons, as an apparent decrease in protein expression was observed in this condition compared to both the siRNA B, blank and scramble conditions (Figure 5a). No difference in NT5C2 protein expression was observed between the blank or scramble conditions, indicating the specificity of the siRNA treatment.

We knocked down *Nt5c2* in DIV15 rat primary cortical neurons and observed the effects on ZFP804A puncta in dendrites and synaptic regions of DIV20 rat primary cortical neurons. The knockdown significantly reduced the number of ZFP804A puncta in synaptic regions—puncta per 10 μm; Blank, 13.28 ± 1.34; Scramble, 12.47 ± 0.48; *Nt5c2* siRNA A, 6.91 ± 2.43. Two‐way ANOVA: *F*(2, 12) = 6.07, *p* = 0.0151; Bonferroni post hoc test (Blank/scramble, *p* = 0.0113, *n* = 3; Scramble/siRNA A, *p* = 0.026, *n* = 3) (Figure 5b+c). Similar to the knockdown, no effect on ZFP804A puncta was observed in dendrites when *Nt5c2* was knocked down. These data suggest that a bidirectional signalling interaction occurs between NT5C2 and ZFP804A, which operate in concert at synapses but not in dendrites.

## 3 DISCUSSION

Mutations in multiple essential synaptic genes have been previously associated with schizophrenia pathogenesis (Forrest et al., 2018; Penzes et al., 2011; Trubetskoy et al., 2022), but the precise nature of how these molecules interact to impair synapses in schizophrenia is unknown. *ZNF804A* and *NT5C2* are robust schizophrenia susceptibility genes (Ripke et al., 2020) that have been previously suggested to interact (Zhou et al., 2018). In this context, ZNF804A has been previously characterised as mediating activity‐dependent structural plasticity of dendritic spines, neurite outgrowth, protein translation and gene transcription (Deans et al., 2017; Hill et al., 2012; Zhou et al., 2018). NT5C2, in turn, is a nucleotidase that regulates AMPK signalling and has putative effects on protein translation (Duarte et al., 2019). However, the molecular mechanisms underlying their association with schizophrenia, or how they relate to each other and impact neuronal/synaptic function, remains unclear. Here, we show that ZFP804A and NT5C2 colocalise and localise to dendritic and synaptic compartments in cortical neurons and that they are part of a protein complex that can bi‐directionally regulate itself, whereby the expression of one protein regulates the expression of the other. This furthers our understanding of these proteins' role in neurons and may contribute to our understanding of how and whether both proteins contribute to synaptic dysfunction in schizophrenia.

This study demonstrates an overlapping subcellular distribution of ZNF804A and NT5C2 in cortical neurons with both proteins being found to be present in multiple subcellular compartments. Consistent with our previous work (Deans et al., 2017), ZFP804A was present in cytosolic and crude membrane/synaptic fractions from cortical neurons. Interestingly, ZFP804A was predominately present in the supernatant fraction of crude membrane/synaptic fractions following detergent separation. This indicates that ZFP804A is transiently, or ‘loosely’, associated with the synaptic membrane. NT5C2 was also present in both cytosolic and crude membrane/synaptic fractions; however, this protein was more abundant in the cytosolic fraction. In crude membrane/synaptic fractions, NT5C2 was also abundant in the supernatant fraction of crude membrane/synaptic fractions following detergent separation. The similar distributions of both proteins were further reflected by immunostaining. Our assessment of ‘synaptic regions’ by immunostaining and our subcellular fractionation protocol does not distinguish protein from being present in both pre‐and post‐synaptic compartments. It includes cytosolic compartments not present in crude membrane/synaptic fractions, as prepared in our study. Nevertheless, consistent with our biochemical analysis, both ZFP804A and NT5C2 were present in the dendritic shaft and within the dendritic spines of eGFP‐expressing neurons. Using super‐resolution imaging, ZFP804A has previously been localised to pre‐synaptic terminals as well as post‐synaptic dendritic spines (Deans et al., 2017). Interestingly, we found that both ZFP804A and NT5C2 co‐localised with the pre‐synaptic marker VGluT1, suggesting that both proteins are present at excitatory synapses. Thus, both proteins may be present in pre‐ and post‐synaptic compartments. Furthermore, we found that NT5C2 and ZFP804A colocalise in regions surrounding the dendrite and that these proteins are part of a protein complex based on co‐immunoprecipitation data. Hence, it is possible that these two proteins functionally interact at or near synapses.

Another main finding of this study is that ZNF804A and NT5C2 form a protein complex. We show that exogenous ZNF804A and NT5C2 form a protein complex in HEK293T cells. Further, we demonstrate that their rodent homologues, ZFP804A and NT5C2, are part of the same complex in primary cortical neurons in dendritic and crude synaptic regions. However, the consequence of this interaction is unclear. As ZNF804A function has been repeatedly linked to dendritic spine maintenance (Deans et al., 2017; Dong et al., 2021) as well as activity‐dependent cytoskeletal restructuring upon chemical long‐term potentiation (cLTP) stimulation (Deans et al., 2017), it would be interesting to test whether NT5C2 may also play a role in these functions through an interaction with ZNF804A. There is a growing body of the literature indicating that cytoskeletal remodelling during spine formation is dependent on local translational processes. Interestingly, both proteins studied here have been shown to regulate protein translation (Duarte et al., 2019; Zhou et al., 2018). Thus, dysregulations of a ZNF804A/NT5C2 protein‐complex may lead to dendritic spine pathology by malfunction of local protein translation. This would be in line with the notion that dendritic spine pathology is a hallmark symptom in post‐mortem brains of schizophrenia patients (Forrest et al., 2018; Penzes et al., 2011). As schizophrenia patient‐derived olfactory neuronal cells and induced pluripotent stem cell‐derived forebrain neural progenitor cells from schizophrenia patients have also been shown to exhibit dysregulated protein synthesis mechanisms (English et al., 2015; Topol et al., 2015), common genetic variants in *ZNF804A* or *NT5C2* could be at least partly responsible for the dysregulation in protein translation converging on dendritic spine pathology and therefore synaptic dysfunction observed in association with schizophrenia. Future studies are warranted to explore if a ZNF804A/NT5C2 protein complex could influence local protein translation and if this influences synaptic and dendritic spine function and maintenance.

A limitation of our study is that, although the knockdown of either *Zfp804a* or *Nt5c2* in primary cortical neurons resulted in a loss of either protein, specifically from synaptic regions, it is challenging to determine whether this was caused by the removal of the protein from synapses or due to a loss of the synapses altogether. For example, the siRNA‐mediated knockdown of *Zfp804a* results in the loss of dendritic spines (Deans et al., 2017). Therefore, the apparent reduction of NT5C2 in synaptic regions could be driven by the fact that there are fewer dendritic spines for the protein to localise to. NT5C2 can signal through AMPK; activation of this kinase has been shown to induce dendritic spine loss (Mairet‐Coello et al., 2013). Thus, the knockdown of *Nt5c2* could induce dendritic spine loss through AMPK activation. There is no evidence of a reduction in the expression of either gene in microarray assays of previously published work where either *ZNF804A* (Hill et al., 2012) or *NT5C2* (Duarte et al., 2019) were knocked down using the same siRNA probes as in this study. This may suggest that the reduction at the protein level may be due to dysregulation of mRNA processing/stability or translation. Indeed, ZNF804A has been linked with the regulation of protein synthesis (Deans et al., 2017; Zhou et al., 2018) and NT5C2 with both protein translation and mRNA decay (Duarte et al., 2019). Further studies are required to understand better how each protein regulates the expression of the other and to clarify the role of NT5C2 and ZNF804A proteins at synapses.

There is a growing appreciation of the need to understand how genetic risk factors interact with each other and environmental factors. It is posited that such interactions likely shape and contribute to complex genetic disorders such as schizophrenia. Recent studies have begun to dissect how different genetic risk factors can synergistically converge on standard biological processes to increase the risk for psychiatric disorders (Schrode et al., 2019). This study demonstrates that two genetic risk factors for psychiatric disorders co‐localise and form a protein complex at synapses, thus revealing a novel understanding of these schizophrenia susceptibility genes. While additional research is required to understand the function of a ZNF804A and NT5C2 protein complex, these data support a model whereby genetic risk factors may directly work in a cooperative or non‐additive manner to increase the risk for schizophrenia.

## 4 METHODS

### 4.1 Cell culture

Human embryonic kidney (HEK293T) cells were maintained in a 37°C/5% CO_2_ atmosphere in DMEM: F12 (Sigma, D6421) supplemented with 10% foetal bovine serum (ClonTech, 631107), 1% L‐glutamine (Sigma, G7513) and 1% penicillin/streptomycin (Life Technologies, 15070063). Cells were passaged and plated at 30%–40% confluency on 1.5H 18 mm glass coverslips 24 h before transfection.

Primary cortical neuronal cultures were harvested from pregnant Sprague–Dawley rats at embryonic day 18 (Srivastava et al., 2011). Animals were habituated for 3 days before experimental procedures, which were carried out in accordance with the Home Office Animals (Scientific procedures) Act, UK, 1986. Cells were seeded on 1.5H 13 mm glass coverslips coated with poly‐D‐lysine 10 μg/mL (Sigma, P0899) diluted in 1× borate buffer (Thermo Scientific, 28341) at a density of 3 × 10^5^/well equating to 857/mm^2^. Cells were cultured in feeding media: neurobasal medium (21103049) supplemented with 2% B27 (17504044), 0.5 mM glutamine (25030024) and 1% penicillin/streptomycin (15070063) (all reagents from Life Technologies, UK). After 4 days in vitro (DIV), 200 μM of d,l‐amino‐phosphonovalerate (d,l‐APV, ab120004; Abcam) was added to media to maintain neuronal health over long‐term culture and to reduce cell death due to excitotoxicity (Srivastava et al., 2011). Fifty percent media changes were performed twice weekly until the desired time in culture was reached (DIV20), at which point cells were transfected or lysed.

### 4.2 Transfection

HEK293T cells and primary cortical neurons were transfected using Lipofectamine 2000 (Life Technologies, 11668019). Briefly, 3 μL lipofectamine 2000 was mixed with 1 μg of each GFP‐ZNF804A (Origene, RG211363) or Myc‐NT5C2 (Origene, RC200194) plasmid construct in DMEM:F12 (Sigma, D6421) or neurobasal medium (primary cortical neurons only). DNA:lipofectamine mixtures were incubated in a 37°C/5% CO_2_ atmosphere for 20 min before being added dropwise to plated HEK293T cells or primary cortical neurons. Transfected HEK293T cells were then further incubated in a 37°C/5% CO_2_ atmosphere for 24–48 h before being fixed for immunocytochemistry or lysed for immunoblotting accordingly. Transfected primary cortical neurons were incubated in a 37°C/5% CO_2_ atmosphere overnight during transfection and then transferred back to feeding media the following day to maintain their viability before being fixed for immunocytochemistry or lysed for immunoblotting accordingly 24 h later.

Short interfering RNA (siRNA) mediated knockdown of *Zfp804a* (the rat homolog of *ZNF804A*) in rat primary cortical neurons was conducted using the N‐Ter Nanoparticle siRNA Transfection System (Sigma: N2788) at DIV15 per manufacturer's instructions. The siRNA targeted exon 2 of *Zfp804a*, and all results were compared to Blank (N‐TER, no transfection) and Scramble (negative control) conditions. Briefly, neurobasal feeding media was removed and replaced with neurobasal transfection media (containing all feeding media components but without penicillin/streptomycin) with 1× APV before transfection. For 3 mL media (1 mL per condition), the following concentrations were required: 14.04 μL of siRNA dilution buffer (Sigma: N0413) was added to 1 μL double‐stranded deoxyribonucleic acid (dsDNA): Blank (N‐TER), Scramble (negative control GC duplex, Thermo Scientific: 465372) or siRNA (Thermo Scientific: HSS150613). In a separate mixture, 7.2 μL N‐TER was mixed with 37.8 μL nuclease‐free water. The transfection mixture and individual siRNA conditions were vortexed for 30 s and spun down with a microcentrifuge. Finally, 15 μL N‐TER transfection mixture was added to each condition, vortexed, spun down and kept in the dark for 20 min at room temperature. Each condition was added dropwise to the relevant coverslips and incubated at 37°C/5% CO_2_ for 5 days before immunocytochemistry.

siRNA‐mediated knockdown of *Nt5c2* in rat primary cortical neurons was conducted using the Trilencer 27‐mer NT5C2 siRNA kit (Origene: SR307908) at DIV15 per manufacturer's instructions. Briefly, primary cortical neurons were fed with 1× d,l‐APV in neurobasal feeding media for 30 min prior to transfection. SiRNA A (sense sequence, 5′‐UGAGAAGUAUGUAGUCAAAGAUGGA‐3′) and siRNA B (sense sequence, 5′‐ACAACUGUAAUAGCUAUUGGUCUTC‐3′) conditions were compared to Blank (N‐TER, no transfection) and Scramble (negative control sense sequence, 5′‐CGUUAAUCGCGUAUAAUACGCGUAT‐3′). N‐TER was mixed with Opti‐MEM (Life Technologies: 31985‐047) at a 1:50 ratio per the manufacturer's instructions. Mixtures were vortexed and spun down using a microcentrifuge and then kept in the dark for 30 min at room temperature. Each mixture was added dropwise to the relevant coverslips and incubated at 37°C/5% CO_2_ for 5 days before immunocytochemistry.

### 4.3 Immunocytochemistry and microscopy

Rat primary cortical neurons and HEK293T cells were fixed in 4% sucrose and 4% formaldehyde for 10 min with agitation. Coverslips were washed in phosphate‐buffered saline (PBS) and then incubated in ice‐cold methanol for 10 min. Next, cells were permeabilized in 0.1% Triton‐X100 (Alfa Aesar, A16046) in PBS and blocked simultaneously in 2% normal goat serum (NGS; Cell Signaling Technology, 5425) in PBS for 1 h with agitation. Coverslips were incubated with primary antibodies diluted in 2% NGS + PBS in a humidified chamber overnight at 4°C. The following day, coverslips were washed 3× in PBS for 15 min each and were incubated with secondary antibodies diluted in 2% NGS + PBS in a humidified chamber at room temperature for 1 h. Coverslips were washed 3× in PBS for 15 min each. Lastly, cells were counterstained using 4′,6‐diamidino‐2‐phenylindole (DAPI; Life Technologies, D1306) diluted in PBS before being mounted on microscope slides with ProLong Gold Antifade Mountant (Life Technologies, P36930) and left to dry for 48 h prior to confocal imaging. Primary antibodies included anti‐ZNF804A C2C3 rabbit polyclonal (GeneTex, GTX121178, 1:200), anti‐NT5C2 mouse monoclonal (Abnova, H00022978‐M02, 1:200), anti‐NT5C2 rabbit polyclonal (Abcam, ab96084, 1:750), anti‐MAP 2 chicken polyclonal (Abcam, ab92434, 1:1000), anti‐VGLUT1 mouse monoclonal (Antibodies Inc., 75066, 1:200), anti‐VGLUT1 rabbit polyclonal (abcam, ab77822, 1:1000), anti‐GFP rabbit polyclonal (Origene, TA150032, 1:10,000) and anti‐Myc mouse monoclonal (BioLegend, 626802, 1:1000). Actin filaments were stained using ActinGreen™ 488 (Life Technologies, R37110) or ActinRed™ 555 (Life Technologies, R37112) ReadyProbes™ per manufacturer's instructions. Secondary antibodies included Alexa Fluor 488 goat anti‐rabbit (A11034, 1:750), Alexa Fluor 568 goat anti‐mouse (A11031, 1:750), Alexa Fluor 633 goat anti‐guinea pig (A21105, 1:500), Alexa Fluor goat anti‐chicken (A21449, 1:750) (all Invitrogen) and Alexa Fluor 405 goat anti‐chicken (Abcam, ab175675, 1:500).

All experiments were imaged using a Leica SP‐5 confocal microscope using a Plan‐Apochromatic 63× 1.40 NA oil‐immersion objective (Leica microsystems, 506210). Fluorophores were excited using a 100 mW Ar laser (458, 476, 488, 496 and 514 nm lines), 10 mW Red He/Ne (633 nm) and 50 mW 405 nm diode laser. Z‐stacks of each cell were acquired for all conditions at 0.5 μm step size using Leica Application Suite–Advanced Fluorescence software (LAS‐AF; v2.7.3) installed on a corresponding desktop computer running Windows XP (Microsoft, SP3). All Z‐stacks were exported to ImageJ (https://imagej.nih.gov/ij/v1.51j8), where maximum intensity projections and background‐subtracted images were generated (Schneider et al., 2012).

### 4.4 Co‐immunoprecipitation, cell fractionation and immunoblotting

HEK293T cells transfected with GFP‐ZNF804A and Myc‐NT5C2 were lysed in co‐immunoprecipitation (co‐IP) buffer consisting of 10 mM Tris; pH 7.4, 150 mM NaCl, 1% Triton‐X100, 0.1% SDS, 1% Deoxycholate, 5 mM EDTA combined with a protease/phosphatase inhibitor cocktail consisting of 1 mM AEBSF, 10 μg/mL Leupeptin, 1 μg/mL Pepstatin A, 2.5 μg/mL Aprotinin, 0.5 M NaF and 1% serine/threonine phosphatase inhibitor cocktail #3 (Sigma, P0044). Detergent soluble lysates were sonicated for 10 pulses at 35% power and centrifuged at 15,000 rpm for 15 min at 4°C to remove cell debris. Samples were then placed on ice for 30 min. An input sample of 75 μL was removed from the lysate, and protein concentration was assessed using a Pierce Bicinchoninic acid (BCA) protein assay kit (Thermo Scientific, 10678484). Concomitantly, 50 μL magnetic Dyna Beads Protein A (Invitrogen, 10002D) were washed in 500 μL tris buffered saline + 0.05% NP40 wash buffer containing 8 μL ZNF804A antibody or 8 μL control Rabbit immunoglobulin (IgG) (Santa Cruz Biotechnology, sc‐2027). Samples were incubated at 4°C for 3 h on a rotator before being placed in a DynaMag™‐2 magnetic rack (Thermo Scientific, 12321D) and washed twice with TBS + NP40 wash buffer. Total lysate (150–200 μL) was equally added to ZNF804A or control normal rabbit IgG‐bound beads. The samples were then left rotating overnight at 4°C. The following day, co‐IP samples were returned to the magnetic rack, and 50 μL of flow‐through was removed and underwent BCA analysis to determine protein concentration. The beads were washed in TBS + NP40 wash buffer and resuspended in 50 μL Laemmli sample buffer (Bio‐Rad Laboratories, 161‐0737), which were subsequently boiled at 95°C for 5 min to denature.

Cell fraction samples were prepared as follows. Cortical tissue from four 16‐week‐old male CD1 mice was homogenized in 10× v/w of homogenisation buffer (0.32 M sucrose, 1 mM NaHCO_3_, 1 mM MgCl_2_) using a glass Dounce tissue homogenizer for 10 strokes. Samples were then centrifuged at 4°C and 200 RCF for 5 min: The nuclear fraction (P1) was discarded, and the supernatant containing the extranuclear cell fraction (S1) was retained. An aliquot of the S1 fraction was then centrifuged at 14,000 RCF for 15 min to produce the cytosolic fraction (S2, supernatant) and crude synaptosomes (P2, pellet) fractions. Next, the P2 pellet was resuspended in 1% Triton X‐100 buffer (20% v/v Triton X‐100) with inhibitors. A further aliquot from the resuspended P2 fractions was centrifuged at 14,000 RCF for 15 min: Supernatant contained the ‘lightly bound’ synaptic fraction (P2S); the pellet was resuspended in homogenisation buffer and contained the ‘tightly bound’ synaptic fraction (P2P). All samples were then stored at −80°C.

Samples were resolved by SDS‐PAGE, transferred to a PVDF membrane and blocked for 1 h in 5% bovine serum albumin (Sigma, A7906) in TBS+0.01% Tween20. Membranes were immunoblotted with primary antibodies overnight at 4°C, followed by 3× 15‐min washes in TBS‐T. Membranes were then incubated with anti‐mouse (Life Technologies, A16078, 1:10,000) or anti‐rabbit (Life Technologies, G‐21234, 1:10,000) horseradish peroxidase (HRP) conjugated secondary antibodies for 1 h at room temperature, followed by 3× 15‐min washes in TBS‐T. Membranes were then incubated in Pierce electrochemiluminescence substrate (Thermo Scientific, 170‐5061) for 5 min and subsequently scanned using a Bio‐Rad ChemiDoc MP (Bio‐Rad). Using Image Lab software (Bio‐Rad, v6.0.1), the band intensity was quantified by densitometry.

### 4.5 Quantification and statistical analysis

Puncta intensities and density were measured on at least two secondary or tertiary MAP 2 positive dendrites, to tally 100 μm in length per neuron. Areas of the dendrite and synaptic region (2 μm on either side of the dendrite) were traced and underwent thresholding, whereby puncta sizes only ranging from 0.08 to 2 μm^2^ were selected for high throughput analysis (Figure 2b). Images of three neurons per condition per culture were analysed of six biological replicate cultures. Puncta count within the dendritic region was compared to puncta count in the synaptic region in synaptic localisation, synaptic co‐localisation and knockdown experiments. All images were background subtracted to exclude non‐specific staining in ImageJ before quantification. Pearson's and Mander's coefficients to assess co‐localisation was performed using the JACoP plugin for ImageJ. To determine the extent of co‐localisation within dendritic and synaptic regions, regions of interests as defined by puncta (as described above) were placed into image channels of the reciprocal protein and the intensity of immunofluorescence measured. Only puncta with immunofluorescence intensities greater than background fluorescence staining were considered an indication of positive co‐localisation. Background fluorescence staining was determined as the average background intensity from five independent regions within a channel, plus two standard deviations from the intensity.

All datasets were subjected to outlier detection using the ROUT method (robust regression and outlier removal; Motulsky & Brown, 2006) in GraphPad Prism (v8.0.2, Windows, GraphPad Software, San Diego, California, USA, http://www.graphpad.com), and detected outliers were removed from their corresponding dataset. All datasets were also tested for normality using the D'Agostino and Pearson normality test to identify which datasets required parametric or non‐parametric analyses (D'agostino et al., 1990). All statistical analyses used an alpha level of 0.05. Non‐parametric Mann–Whitney *U* test was used to analyse all synaptic localisation and synaptic co‐localisation data. Ordinary one‐way analysis of variance (ANOVA) with Bonferroni correction for multiple comparisons was used for analysing ZFP804A puncta in *Zfp804a* knockdown experiments. Two‐way ANOVA with Tukey's test for multiple comparisons was used for analysing the effects of *Zfp804a* knockdown on NT5C2 puncta. Two‐way ANOVA with Tukey's test for multiple comparisons was also used for analysing the effects of *Nt5c2* knockdown on ZFP804A puncta. All data are shown as mean ± standard error of the mean (SEM), and all error bars represent SEM.

## AUTHOR CONTRIBUTIONS

Afra Aabdien, Laura Sichlinger, Zoe Borgel, Iain A. Waston, Nicholas J. F. Gatford, Pooja Raval, Madeleine R. Jones, Lloyd Tanangonan and Deepak P. Srivastava performed all experiments and subsequent analysis. Deepak P. Srivastava, Timothy R. Powell and Rodrigo R. R. Duarte designed the project and experiments. Afra Aabdien, Nicholas J. F. Gatford and DPS wrote the initial draft of the manuscript; Afra Aabdien, Laura Sichlinger, Nicholas J. F. Gatford, Pooja Raval, Madeleine R. Jones, Lloyd Tanangonan, Timothy R. Powell, Rodrigo R. R. Duarte and Deepak P. Srivastava edited and finalized the manuscript.

## CONFLICT OF INTEREST STATEMENT

The authors declare that they have no conflict of interest.

### PEER REVIEW

The peer review history for this article is available at https://www.webofscience.com/api/gateway/wos/peer-review/10.1111/ejn.16254.

## Supporting information


**Figure S1.** Workflow for isolating cytosolic and crude synaptosomal fractions from mouse cortex. S1 = extranuclear fraction; S2 = cytosolic fraction; P2 crude membrane/synaptic fraction; P2S – soluble fraction (supernatant) following detergent extraction = P2S; insoluble fraction (precipitate) following detergent extraction = P2P.
**Figure S2. (A)** Representative confocal image of a section of dendrite from DIV20 cortical neuron expressing eGFP (morphological marker) and co‐stained for ZFP804A and VGluT1. Co‐localisation of ZFP804A with VGluT1 could be observed and was confirmed by conducting orthogonal view analysis with VGluT1, whereby the 2D immunocytochemistry could be viewed and superimposed in 3D by producing an X, Y and Z plane. **(B)** Representative confocal image of a section of dendrite from DIV20 cortical neuron expressing eGFP (morphological marker) and co‐stained for NT5C2 and VGluT1. Co‐localisation of NT5C2 with VGluT1 could be observed and was confirmed by conducting orthogonal view analysis with VGLuT1, whereby the 2D immunocytochemistry could be viewed and superimposed in 3D by producing an X, Y and Z plane. Scale bar = 10 μm.
**Figure S3:** Representative confocal images of DIV20 rat primary cortical neurons overexpressing Myc‐NT5C2, immunostained for morphological marker MAP 2 (blue) and Myc (green).

## Data Availability

Primary data material can be accessed by contacting the corresponding authors.
