# Supplementary material for: Schizophrenia risk proteins ZNF804A and NT5C2 interact in cortical neurons
Source: Eur J Neurosci. 2024 Jan 26;59(8):2102–17. doi: 10.1111/ejn.16254 (PMC11170667; doi:10.1111/ejn.16254)
Supplement: Supplementary file 1 — Figure S1. Workflow for isolating cytosolic and crude synaptosomal fractions from mouse cortex. S1 = extranuclear fraction; S2 = cytosolic fraction; P2 crude membrane/synaptic fraction; P2S – soluble fraction (supernatant) following detergent extraction = P2S; insoluble fraction (precipitate) following detergent extraction = P2P. Figure S2. (A) Representative confocal image of a section of dendrite from DIV20 cortical neuron expressing eGFP (morphological marker) and co‐stained for ZFP804A and VGluT1. Co‐localisation of ZFP804A with VGluT1 could be observed and was confirmed by conducting orthogonal view analysis with VGluT1, whereby the 2D immunocytochemistry could be viewed and superimposed in 3D by producing an X, Y and Z plane. (B) Representative confocal image of a section of dendrite from DIV20 cortical neuron expressing eGFP (morphological marker) and co‐stained for NT5C2 and VGluT1. Co‐localisation of NT5C2 with VGluT1 could be observed and was confirmed by conducting orthogonal view analysis with VGLuT1, whereby the 2D immunocytochemistry could be viewed and superimposed in 3D by producing an X, Y and Z plane. Scale bar = 10 μm. Figure S3: Representative confocal images of DIV20 rat primary cortical neurons overexpressing Myc‐NT5C2, immunostained for morphological marker MAP 2 (blue) and Myc (green). [file EJN-59-2102-s001.pdf]

Schizophrenia Risk Proteins ZNF804A and NT5C2 Interact in cortical neurons

Afra Aabdien[1,2], Laura Sichlinger[1,2], Zoe Borgel[1,2], Madeleine R. Jones[1,2], Iain A. Waston[1,2], Nicholas J.F. Gatford[1,2], Pooja Raval[1,2], Lloyd Tanangonan[1,2], Timothy R. Powell[3], Rodrigo R.R. Duarte[3], Deepak P. Srivastava[1,2]*

Supplemental Information:

Supplemental Figures 1-3

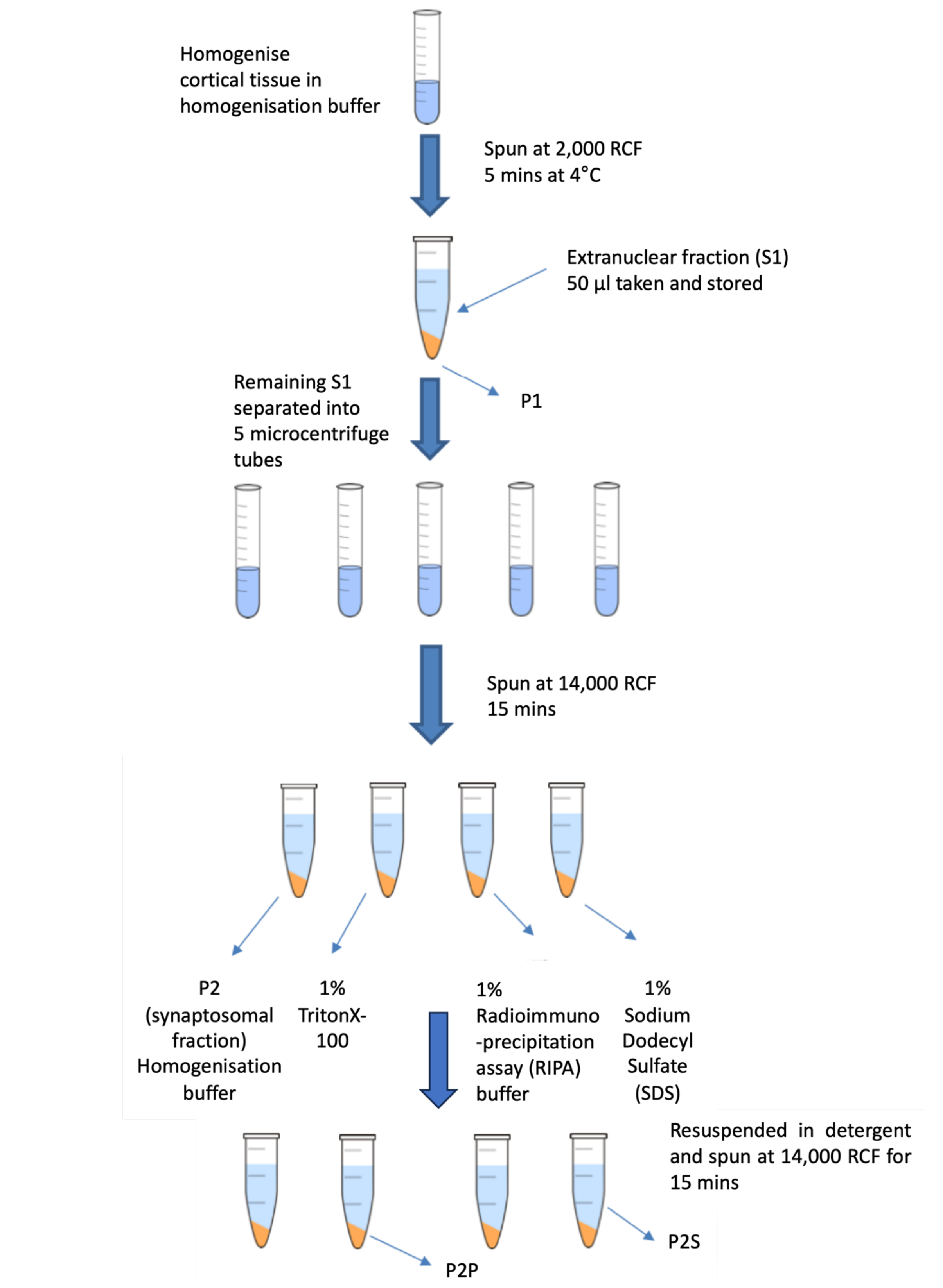

Supplemental Figure 1. Workflow for isolating cytosolic and crude synaptosomal fractions from mouse cortex. S1 = extranuclear fraction; S2 = cytosolic fraction; P2 crude membrane/synaptic fraction; P2S – soluble fraction (supernatant) following detergent extraction = P2S; insoluble fraction (precipitate) following detergent extraction = P2P

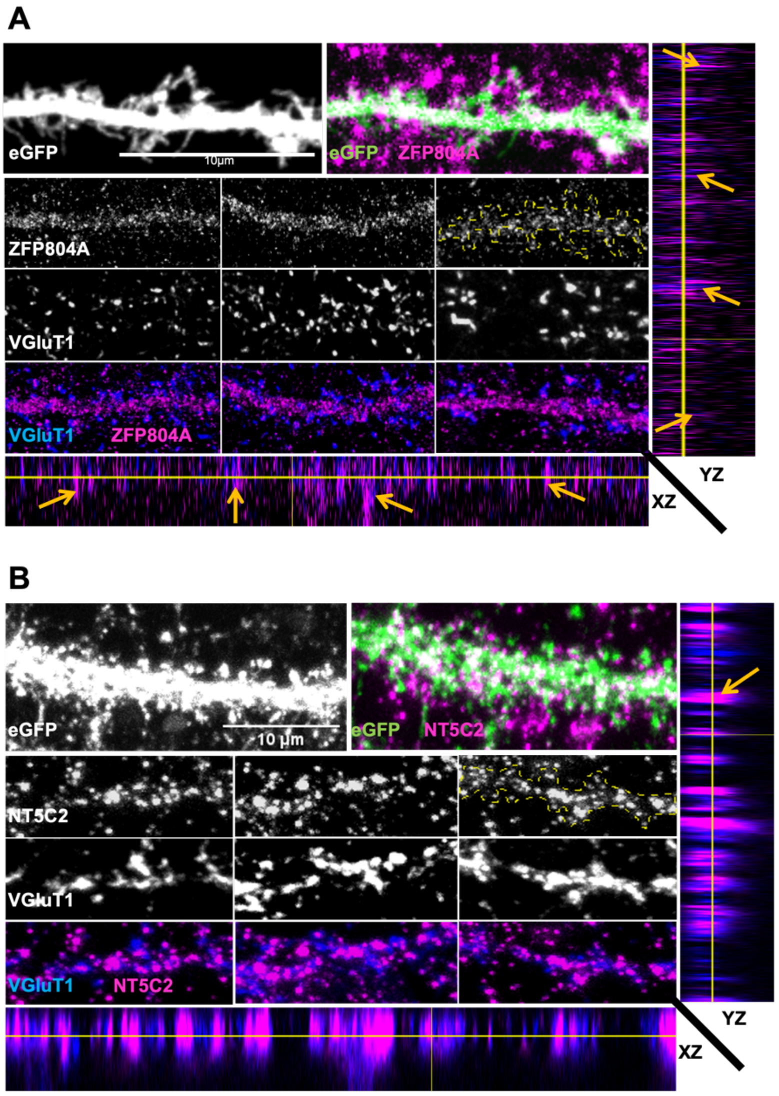

Supplemental Figure 2. (A) Representative confocal image of a section of dendrite from DIV20 cortical neuron expressing eGFP (morphological marker) and co-stained for ZFP804A and VGluT1. Co-localisation of ZFP804A with VGluT1 could be observed and was confirmed by conducting orthogonal view analysis with VGluT1, whereby the 2D immunocytochemistry could be viewed and superimposed in 3D by producing an X, Y and Z plane. **(B)** Representative confocal image of a section of dendrite from DIV20 cortical neuron expressing eGFP (morphological marker) and co-stained for NT5C2 and VGluT1. Co-localisation of NT5C2 with VGluT1 could be observed and was confirmed by conducting orthogonal view analysis with VGLuT1, whereby the 2D immunocytochemistry could be viewed and superimposed in 3D by producing an X, Y and Z plane. Scale bar = 10 µm.

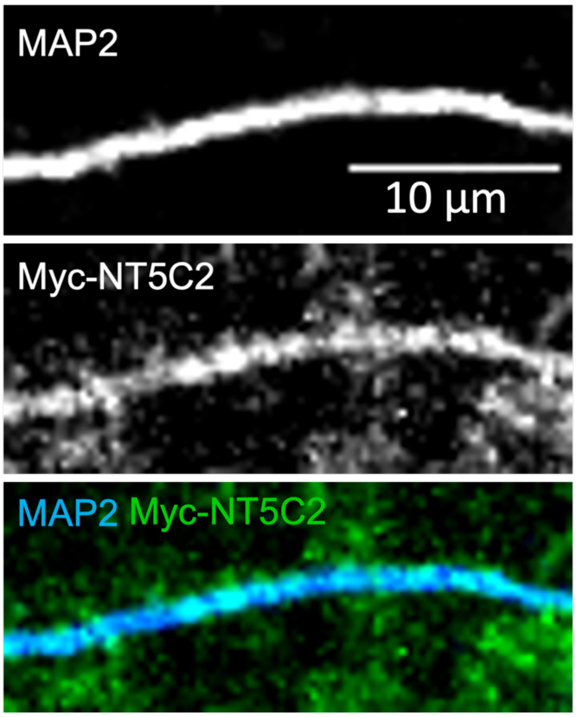

Supplementary figure 3: Representative confocal images of DIV20 rat primary cortical neurons overexpressing Myc-NT5C2, immunostained for morphological marker MAP2 (blue) and Myc (green).
